# The Molecular Tumor Board Portal supports clinical decisions and automated reporting for precision oncology

**DOI:** 10.1038/s43018-022-00332-x

**Published:** 2022-02-24

**Authors:** David Tamborero, Rodrigo Dienstmann, Maan Haj Rachid, Jorrit Boekel, Adria Lopez-Fernandez, Markus Jonsson, Ali Razzak, Irene Braña, Luigi De Petris, Jeffrey Yachnin, Richard D. Baird, Yohann Loriot, Christophe Massard, Patricia Martin-Romano, Frans Opdam, Richard F. Schlenk, Claudio Vernieri, Michele Masucci, Xenia Villalobos, Elena Chavarria, Cancer Core Europe consortium, Shubha Anand, Danny Baars, Svetlana Bajalica-Lagercrantz, Richard Baird, Mariska Bierkens, Lennart Blomqvist, Costanza Bono, Luigi De Petris, Gary J. Doherty, Arnauld Forest, Valentina Fornerone, Paola Gabaldi, Felix Haglund, Johan Hartman, Peter Horak, Tanja Jutzi, Mary Kasanicki, Simon Kreutzfeldt, Lucian Le Cornet, Rolf Lewensohn, Johan Lindberg, Carlos Lopez, Andreas Lundqvist, Jose-Ezequiel Martin, Gerrit Meijer, Susana Muñoz, Maud Ngo Camus, Claudio Nicotra, Paolo Nuciforo, Petra Oberrauch, Päivi Östling, Laura Pelz, Alejandro Piris-Gimenez, Elena Provenzano, Etienne Rouleau, John Rowell, Omar Saavedra, Giovanni Scoazec, Kenneth Seamon, Marc Tischkowitz, Lizet van der Kolk, Ruud van der Noll, Maria Vieito, Daniel Vis, Ana Vivancos, Christina von Gertten, Anders Wennborg, Lodewyk Wessels, Valtteri Wirta, Judith Balmaña, Giovanni Apolone, Carlos Caldas, Jonas Bergh, Ingemar Ernberg, Stefan Fröhling, Elena Garralda, Claes Karlsson, Josep Tabernero, Emile Voest, Jordi Rodon, Janne Lehtiö

**Affiliations:** 1 Department of Oncology and Pathology, Karolinska Institutet, Science for Life Laboratory, Stockholm, Sweden; 2 Medical Oncology, Oncology Data Science, Vall d’Hebron Institute of Oncology (VHIO), Barcelona, Spain; 3 Hereditary Cancer Genetics Group, Vall d’Hebron Institute of Oncology (VHIO), Barcelona, Spain; 4 Medical Oncology Department, Vall d’Hebron University Hospital, Vall d’Hebron Institute of Oncology (VHIO), Barcelona, Spain; 5 Department of Oncology and Pathology, Karolinska Institutet, Theme Cancer, Karolinska Comprehensive Cancer Center, Karolinska University Hospital, Stockholm, Sweden; 6 Cancer Research UK Cambridge Centre, Cambridge, UK; 7 Département d’Innovation Thérapeutique et d’Essais Précoces, Gustave Roussy, Université Paris-Saclay, Villejuif, France; 8 The Netherlands Cancer Institute, Amsterdam, the Netherlands; 9 NCT Trial Center, German Cancer Research Center, Heidelberg University Hospital, Heidelberg, Germany; 10 Fondazione IRCCS Istituto Nazionale dei Tumori, Milan, Italy; 11 IFOM, FIRC Institute of Molecular Oncology, Milan, Italy; 12 Department of Microbiology, Tumor and Cell Biology, Karolinska Institutet, Stockholm, Sweden; 13 Vall d’Hebron Institute of Oncology (VHIO), Barcelona, Spain; 14 Scientific Directorate, Fondazione IRCCS Istituto Nazionale dei Tumori, Milan, Italy; 15 National Center for Tumor Diseases Heidelberg, German Cancer Research Center, Heidelberg, Germany; 16 Department of Oncology and Pathology, Karolinska Institutet, Stockholm, Sweden; 17 Department of Hematology, Karolinska University Hospital, Stockholm, Sweden; 18 Oncode Institute, Utrecht, the Netherlands; 19 Department of Investigational Cancer Therapeutics, University of Texas MD Anderson Cancer Center, Houston, TX USA; 20 Clinical Proteomics Unit, Department of Oncology and Pathology, Karolinska Institutet, Science for Life Laboratory, Karolinska University Hospital, Stockholm, Sweden; 21 Cancer Molecular Diagnostics Laboratory, Department of Oncology, University of Cambridge, Cambridge, UK; 22 Department of Scientific Administration, The Netherlands Cancer Institute, Amsterdam, The Netherlands; 23 Department of Oncology-Pathology, Karolinska Institutet, Stockholm, Sweden; 24 Department of Clinical Genetics, Karolinska University Hospital, Stockholm, Sweden; 25 Department of Pathology, The Netherlands Cancer Institute, Amsterdam, The Netherlands; 26 Department of Imaging and Physiology, Karolinska University Hospital, Stockholm, Sweden; 27 Department of Molecular Medicine and Surgery, Karolinska Institutet, Stockholm, Sweden; 28 Department of Oncology, Cambridge University Hospitals NHS Foundation Trust, Cambridge Biomedical Campus, Cambridge, UK; 29 Institut Gustave Roussy, Villejuif, France; 30 Department of Oncology-Pathology, Karolinska Institutet, Stockholm, Sweden; 31 Clinical Pathology and Cancer Diagnostics, Karolinska University Hospital, Stockholm, Sweden; 32 Department of Oncology-Pathology, Karolinska Institutet, Stockholm, Sweden; 33 Division of Translational Medical Oncology, National Center for Tumor Diseases Heidelberg and German Cancer Research Center, Heidelberg, Germany; 34 National Institute for Health Research Cambridge Biomedical Research Centre, University of Cambridge, Cambridge, UK; 35 NCT Trial Center, German Cancer Research Center and Heidelberg University Hospital, Heidelberg, Germany; 36 Theme Cancer, Karolinska Comprehensive Cancer Center, Stockholm, Sweden; 37 Department of Oncology-Pathology, Karolinska Institutet, Stockholm, Sweden; 38 Department of Medical Epidemiology and Biostatistics, Science for Life Laboratory, Karolinska Institutet, Stockholm, Sweden; 39 Business Development Area, Vall d’Hebron Institute of Oncology (VHIO), Vall d’Hebron Barcelona Hospital Campus, Barcelona, Spain; 40 Clinical Research Support Unit, Vall d’Hebron Institute of Oncology (VHIO), Vall d’Hebron Barcelona Hospital Campus, Barcelona, Spain; 41 DITEP– Drug Development Department, Institut Gustave Roussy, Villejuif, France; 42 Molecular Oncology Group, Vall d’Hebron Institute of Oncology (VHIO), Vall d’Hebron Barcelona Hospital Campus, Barcelona, Spain; 43 Department of Oncology and Pathology, Science for Life Laboratory, Karolinska Institutet, Stockholm, Sweden; 44 Research Coordination Area, Vall d’Hebron Institute of Oncology (VHIO), Vall d’Hebron Barcelona Hospital Campus, Barcelona, Spain; 45 Tumor Genetic Lab, Institut Gustave Roussy, INSERM UMR 981 Villejuif, France; 46 Cancer Core Europe, Institut Gustave Roussy, Villejuif, France; 47 Medical Oncology Department, Vall d’Hebron Institute of Oncology (VHIO), Hospital Universitari Vall d’Hebron, Vall d’Hebron Barcelona Hospital Campus, Barcelona, Spain; 48 Family Cancer Clinic, The Netherlands Cancer Institute, Amsterdam, the Netherlands; 49 Drug development Unit, Vall d’Hebron Institute of Oncology (VHIO), Barcelona, Spain; 50 Oncode Institute, The Netherlands Cancer Institute, Amsterdam, The Netherlands; 51 Cancer Genomics Group, Vall d’Hebron Institute of Oncology (VHIO), Vall d’Hebron Barcelona Hospital Campus, Barcelona, Spain; 52 Department of Microbiology, Tumor and Cell Biology, Science for Life Laboratory, Karolinska Institutet, Stockholm, Sweden

**Keywords:** Cancer genomics, Cancer genetics, Cancer, Computational biology and bioinformatics

## Abstract

There is a growing need for systems that efficiently support the work of medical teams at the precision-oncology point of care. Here, we present the implementation of the Molecular Tumor Board Portal (MTBP), an academic clinical decision support system developed under the umbrella of Cancer Core Europe that creates a unified legal, scientific and technological platform to share and harness next-generation sequencing data. Automating the interpretation and reporting of sequencing results decrease the need for time-consuming manual procedures that are prone to errors. The adoption of an expert-agreed process to systematically link tumor molecular profiles with clinical actions promotes consistent decision-making and structured data capture across the connected centers. The use of information-rich patient reports with interactive content facilitates collaborative discussion of complex cases during virtual molecular tumor board meetings. Overall, streamlined digital systems like the MTBP are crucial to better address the challenges brought by precision oncology and accelerate the use of emerging biomarkers.

## Abstract (web summary)

Tamborero and colleagues develop the Molecular Tumor Board Portal, a virtual platform that integrates and interprets genomics and clinical data from prospective clinical studies, to support clinical decision-making for precision oncology.

## Main

Next-generation sequencing (NGS) assays are a key component of the modern oncology workflow. Beyond on-label drug prescriptions, tumor sequencing results can guide clinical trial enrollment and identify investigational drug opportunities in individual patients. NGS data can also reveal other events of clinical relevance, such as germline pathogenic variants, pharmacogenomics findings and clonal hematopoiesis drivers, which should be recognized and acted upon. However, clinical interpretation of NGS results often relies on manual procedures, which poses considerable challenges to the medical teams undertaking this task. First, variant annotation benefits from numerous resources developed by medical, biological and bioinformatics domains that are not easy to integrate. Second, agreeing on annotation criteria and rules to prioritize actionable findings is critical for consistent clinical decision-making. Third, in the absence of on-label treatment options, patients must be matched with the specific portfolio of investigational therapies and clinical trials available in each hospital (or hospital network), which are subject to continuous changes. Failure to address these issues or the inability to perform them in a clinically acceptable time frame can impair the outcome of individual patients and precision cancer medicine initiatives.

Clinical decision support systems (CDSS) can tackle these challenges by implementing efficient data analysis and reporting processes. Several commercial CDSS software are currently available, but in-house solutions are often used to better accommodate the specific needs of each center. In fact, we believe that the capacity of academic institutions to develop custom CDSS accelerates the use of emerging biomarkers and promotes precision medicine across healthcare professionals. We have therefore developed the MTBP, a CDSS that creates a unified framework to interpret sequencing results across the seven comprehensive cancer centers that form Cancer Core Europe (CCE)^1^ at present. Importantly, the portal is integrated in the CCE clinical workflows and provides a single platform to distribute the results and support shared discussions at scale^2^. Seamless communication among clinical investigators is essential to leverage the collective expertise of the community in this era of rapidly changing precision-oncology landscape. To our knowledge, this is an unprecedented effort for codeveloping new anticancer therapies and biomarkers under a harmonized infrastructure in Europe. Here, we describe our approach and discuss the results of using the MTBP in a consecutive cohort of 500 advanced solid tumors evaluated from January 2019 to January 2021 in the context of the Basket of Baskets (NCT03767075) study, an ongoing CCE multibasket phase 2 clinical trial matching molecular biomarkers with immunotherapies and targeted drugs.

## Results

### Functional interpretation of cancer variants

**Fig. 1:**
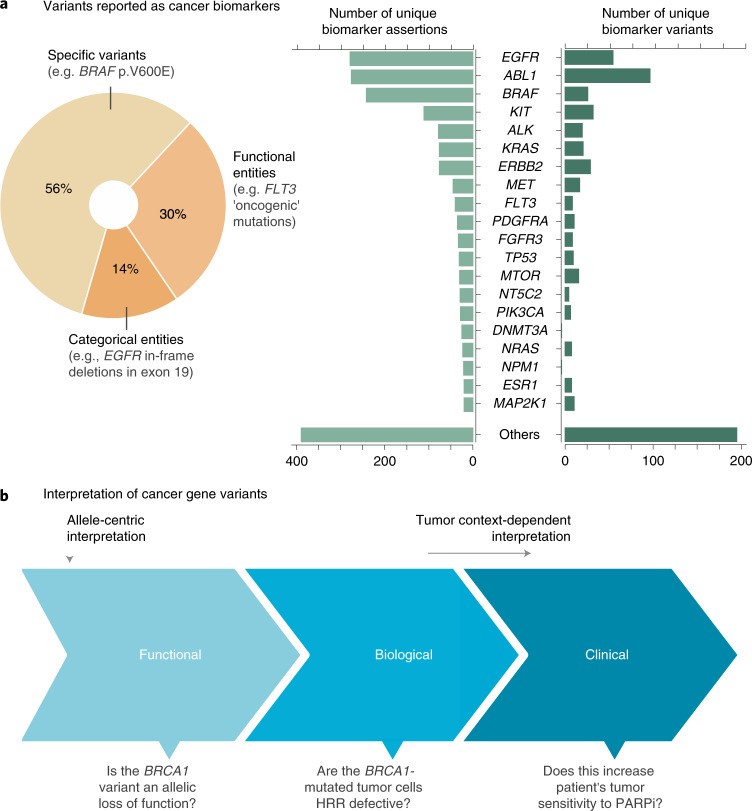
Clinical relevance of cancer gene variants. **a**, Overview of gene mutations (single-nucleotide variants and small indels) reported as biomarkers of cancer diagnosis, prognosis and/or drug response by three publicly available knowledge bases (CIViC (Clinical Interpretation of Variants in Cancer), OncoKB (Oncology Knowledge Base) and CGI (Cancer Genome Interpreter)^6–8^) at the moment of writing. An assertion corresponds to a reported biomarker effect for a given gene variant, in a given cancer type and with a given level of supporting evidence. Assertions supported by weaker or inconclusive evidence (as provided by the knowledge base metadata when appropriate; Methods) are excluded from these results. 1,000g, 1000 Genomes Project; AF, allele frequency. **b**, Representation of distinct levels of interpretation for cancer gene variants. The functional relevance evaluates the allele-centric effect of the observed variant, whereas context-dependent interpretation factors in additional considerations (such as whether the variant is germline or somatic, co-occurring alterations in the same or other genes and/or the cancer type of the patient’s tumor). Questions that are addressed in each step are exemplified here for a given *BRCA1* mutation (HRR, homologous recombination repair; PARPi, poly-ADP ribose polymerase inhibitors). Source data

Interpretation of NGS data first requires elucidating whether the specific variants observed in the tumor alter the wild-type function of cancer genes, as not all of them have equal biological consequences. Besides the identification of the individual tumor genomic drivers, this analysis enables matching patients to biomarkers defined by functional criteria, such as ‘activating’ mutations in a given oncogene or ‘loss-of-function’ alterations in a given tumor suppressor. Of note, close to one-third of the cancer biomarkers reported at present rely on interpreting the functional effect of variants found in drug targets (Fig. 1a). This number will likely continue to grow as genes involved in more cellular processes, such as DNA damage repair, epigenetics, metabolism and immune regulation, become actionable.

**Fig. 2:**
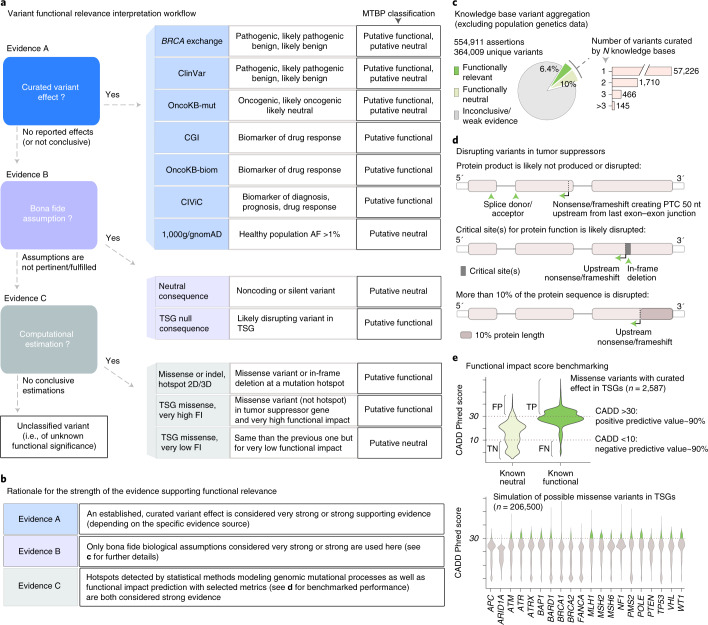
Interpretation of cancer gene variants. **a**, The MTBP classifies a given cancer gene variant as (putative) functionally relevant or neutral according to three distinct sources of evidence (named A, B and C here) or of unknown relevance if none of these criteria are fulfilled. Note that the knowledge bases listed here are those integrated at the moment of writing^4–10^, but their usage may be subject to changes depending on evolving needs and preferences. FI, functional impact; OncoKB-mut and OncoKB-biom refer to the biological and predictive relevance annotation of variants in OncoKB, respectively. **b**, Criteria supporting the variant functional classification are considered to provide strong (>0.9 certainty) or very strong (>0.99 certainty) evidence as extrapolated from the work in variant pathogenicity classification^3^, following the rationale described in the table. **c**, Aggregated knowledge base assertions (excluding those from genetics population data) at the moment of writing. As expected by the different scopes of each knowledge base and the long tail of lowly recurrent mutations, only a minority of the variants appear curated in more than one knowledge base, which stresses the importance of their aggregation to provide a comprehensive annotation. **d**, Graphical summary of some of the criteria used for assuming that a variant with null consequence type is disrupting the function of a given tumor suppressor (part of the evidence of type B; **a**). These are largely based on established rules to identify loss-of-function variants in Mendelian genes (Methods). **e**, The lowest level of evidence to estimate a given variant effect is based on bioinformatics metrics. For variants that are not located in mutation hotspots, we decided to use the combined annotation dependent depletion (CADD) score^16^ to estimate the functional relevance of missense mutations in tumor suppressor genes (TSGs), as functional impact predictions perform worse in other scenarios (data not shown). The method and associated thresholds were selected according to our own benchmarking, based on the performance observed for mutations with curated effects (upper violin plot) and in silico simulations (lower violin plot) (Methods). FN, false negative (given these thresholds); FP, false positive; TN, true negative; TP, true positive. Source data

The MTBP interprets the functional relevance of cancer variants under an allele-centric perspective (Fig. 1b). In other words, a given *BRCA1* mutation known to disrupt the activity of the wild-type allele will always be declared as functionally relevant (i.e., loss of function) regardless of tumor-context considerations such as the germline versus somatic origin of the variant, the status of the second allele and/or the cancer type in which it is observed, which are contemplated in the actionability analysis (next section). Multiple genomic knowledge resources can be integrated for a more comprehensive variant functional annotation, but currently there are no well established guidelines on how to do it. Therefore, we agreed on criteria considered to provide strong or very strong supporting evidence (>90% and >99% of certainty, respectively) as extrapolated from previous work^3^ and based on three distinct sources of knowledge (Fig. 2a,b). First, the MTBP inspects whether the gene variants observed in the patient’s tumor have an already well-reported effect. Note that different types of assertions can be equally mapped to the context-agnostic notion of a variant being functionally relevant; for example, a given *BRCA1* mutation can be considered as a putative loss-of-function event when it is known to predispose to early breast/ovarian cancer, as well as when it is associated with clinical efficacy of poly-ADP ribose polymerase inhibitors. Therefore, the MTBP queries a number of expert knowledge bases that continuously gather results of clinical, experimental and population genetic studies^4–10^ according to the standard procedures defined for each of their respective scopes (e.g., pathogenicity classification of germline variants^11^), and assertions compatible with the functional relevance (or lack thereof) of the observed variant are matched as appropriate. The aggregation of these different knowledge bases, which are not often used in combination, enables a more comprehensive use of international curation efforts (Fig. 2c).

Second, if no variant effect is reported, or the information is inconclusive and/or supported by weaker evidence (Methods), then the portal evaluates whether bona fide biological assumptions (such as whether a given premature termination codon is likely to trigger nonsense-mediated decay) can be applied (Fig. 2d). These assumptions are largely based on accepted criteria to identify loss-of-function variants in Mendelian disease genes^11,12^. Of note, the MTBP refines the use of some of these criteria by leveraging the aggregated content of the aforementioned knowledge bases, which for example help to delineate protein regions that are critical for a given tumor suppressor function (Methods). This exemplifies the value of the MTBP for integrating the knowledge available in the community and developing ensemble bioinformatics models.

Third, if none of these bona fide assumptions can be applied or fulfilled, then computational-based metrics are used as the lowest level of supporting evidence. For example, hotspots of somatic mutations observed across previously sequenced cancer cohorts point out protein sites that are preferentially targeted by tumors and thus relevant for the disease development in both oncogenes and tumor suppressors^13^. To reduce the number of false positives, the MTBP uses methods that consider underlying genomic mutational processes to declare the observed accumulation of mutations as statistically significant^14,15^. In addition, functional impact predictions can be used to estimate whether other variants drive loss-of-function events. Among all the methods developed with that purpose, we decided to use deleteriousness score calculations^16^, with stringent thresholds exhibiting a 90% predictive value as required for strong supporting criteria^3^, based on the results of our own benchmarking (Fig. 2e).

**Fig. 3:**
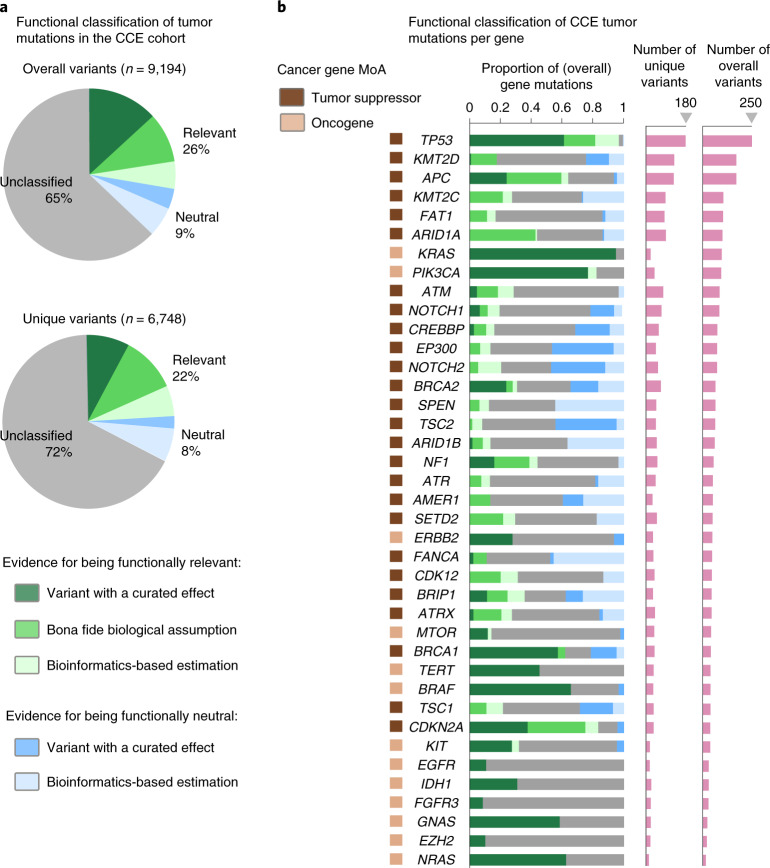
MTBP functional classification of variants in the Cancer Core Europe cohort. **a**, Functional classification of the tumor mutations observed in the CCE cohort (variants assumed to be functionally neutral, such as common polymorphisms, are not included here). Colors represent the different sources of supporting evidence used by the MTBP (Fig. 2). The upper pie chart represents overall counts of mutations, whereas the lower pie chart represents counts of unique mutations. **b**, Functional classification of the tumor mutations (represented as in **a**) observed across several of the most recurrently mutated cancer genes in the CCE cohort. Tumor suppressors tend to accumulate a variety of null events (e.g., frameshift or nonsense variants) whose effect is mainly estimated by bona fide biological assumptions, except for genes that have been more exhaustively characterized by ongoing efforts (e.g., *BRCA1* and *BRCA2*) and/or are enriched by specific dominant-negative mutations (e.g., *TP53* and, to a lesser extent, *PTEN*). In oncogenes, variants are concentrated in few hotspots with a well-known gain-of-function consequence in some genes (e.g., *KRAS* and *BRAF*), whereas others show a diversity of mutations whose effect remains unclassified in a considerable number of cases (e.g., *ERBB2* and *FGFR3*). MoA, mechanism of action. Source data

Variants that cannot be classified as functionally relevant or functionally neutral according to any of the aforementioned criteria appear as ‘of unknown relevance’ in the MTBP reports. For the CCE prospective cohort presented here, composed of 500 solid tumors profiled by NGS panels (from 326 to 350 cancer genes evaluated, depending on the assay; Table 1), the MTBP identified a median of three (interquartile range (IQR), two to four) functionally relevant mutations (single-nucleotide changes and/or small indels) per tumor. Overall, and after excluding mutations assumed to be nonrelevant (such as those that do not alter the protein sequence or are common polymorphisms), a total of 26% of the tumor mutations were classified as (putative) functionally relevant, whereas 9% were classified as (putative) neutral (Fig. 3a). One-fourth of these classifications were solely based on bioinformatic predictions, which as discussed is the lowest level of supporting evidence. Even with the comprehensive functional annotation provided by the MTBP, most (65%) of the tumor mutations observed in cancer genes were thus classified as of unknown functional effect (although this number largely varies across genes; Fig. 3b). This illustrates our still-limited ability for interpreting the biological relevance of the genomic alterations that occur in tumor cells. As drug prescriptions progressively move toward a more holistic consideration of the tumor genome (pathway and/or signature centric), we underscore the importance of using interpretation tools that are kept up to date with the knowledge provided by emerging capabilities, such as high-throughput functional assays.

**Table 1:** Characteristics of the 500 tumors in the CCE prospective cohort

Characteristics	Values
Age (y), median (IQR)	59 (49–67)
Female sex, *n* (%)	300 (60%)
Paired samples^a^, *n* (%)	375 (75%)
Primary tumor sample^b^, *n* (%)	270 (54%)
Tumor purity^c^, median (IQR)	60% (35–80)
Primary cancer type, *n* (%)	
Breast carcinoma	100 (20%)
Colorectal adenocarcinoma	65 (13%)
Ovarian epithelial tumor	55 (11%)
Esophagogastric adenocarcinoma	30 (6%)
Cholangiocarcinoma	25 (5%)
Pancreatic adenocarcinoma	20 (4%)
Cancer of unknown primary	15 (3%)
Prostate adenocarcinoma	10 (2%)
Salivary carcinoma	10 (2%)
Pleural mesothelioma	10 (2%)
Gallbladder cancer	10 (2%)
Other	150 (30%)

Patients with advanced/refractory disease considered for CCE clinical trials from January 2019 to January 2021. Note that the cohort is biased toward those cancer types that were more suited to the Basket of Baskets treatment arms opened during that time period. All samples were profiled by targeted NGS panels.

^a^Sequencing of paired white blood cells and tumor tissue samples identifying germline versus tumor somatic variants; only the tumor sample was sequenced otherwise.

^b^Sequenced tumor sample obtained from the primary tumor; sample was from a metastatic site otherwise.

^c^Percentage of tumor content in the tumor sample as reported by the pathology assessment.

### Clinical interpretation of cancer variants

The final objective of the MTBP is to help translate NGS results into the most appropriate therapeutic decisions according to state-of-the-art evidence. Genomic alterations that influence anticancer drug response (sensitivity or resistance) and are of diagnostic or prognostic value are continuously reported in the literature and scientific venues. Several international initiatives gather this information in specific knowledge bases open for the access and feedback of the community^6–8^. However, these resources follow varying data models, and the accurate aggregation of their content requires an extensive harmonization of the lexicon, ontologies and variant representation syntax used by each. The MTBP implements this process with a semiautomatic pipeline that combines a number of bioinformatic mapping tools^17,18^ and manually annotated dictionaries. The adoption of information exchange standards in the community is crucial to mitigate the need for these efforts and facilitate genomic knowledge sharing^19–21^.

**Fig. 4:**
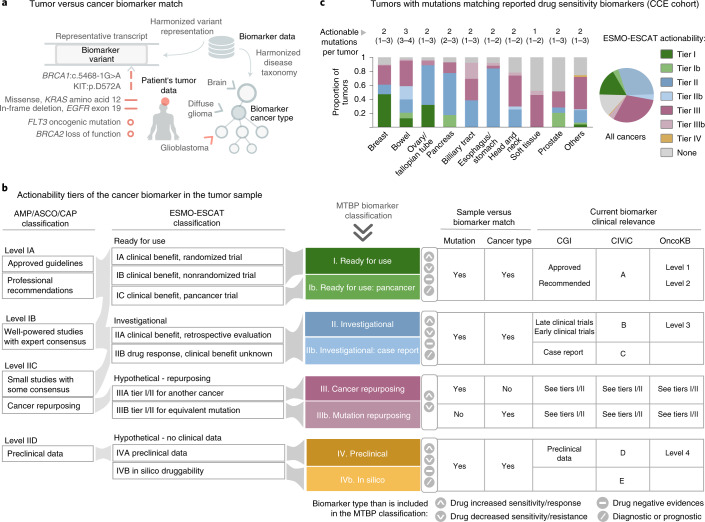
MTBP cancer biomarkers matching in the Cancer Core Europe cohort. **a**, The MTBP matches the observed tumor variants with cancer biomarkers reported as a specific nucleotide or protein change (see examples next to the red vertical lines), a categorical genomic definition (red horizontal line examples) or a functional entity (matched according to the MTBP functional interpretation; red circle examples). Variant actionability must also factor in (among others) the coincidence between the biomarker and patient’s cancer type; the MTBP takes into account the disease hierarchy so the biomarker is matched when reported for the patient’s tumor type or a subtype thereof (red arrow). **b**, The MTBP ranks the cancer biomarkers (diagnosis, prognosis and drug response) found in the tumor following the ESMO/ESCAT scale^22^ with two minor modifications (Methods). An approximate equivalence with the actionability proposed by the Association for Molecular Pathology, American Society of Clinical Oncology and College of American Pathologists (AMP/ASCO/CAP)^23^ is also shown (left). The table summarizes how the MTBP classifies the biomarker actionability according to the coincidence of the (i) variant and (ii) cancer type reported for the patient’s tumor versus biomarker and (iii) the clinical evidence supporting the biomarker effect (as curated by each of the biomarker knowledge bases used by the MTBP at the moment of writing). **c**, Tumor mutations observed in the CCE cohort matching with drug sensitivity biomarkers (biomarkers of resistance or toxicity are not included here). Colors represent the highest level of actionability of all the biomarkers found in each tumor. Numbers above the bars represent the median (percentile 25–75) number of unique mutations per tumor reported as drug sensitivity biomarkers, regardless of their level of actionability. Cancer types are grouped by their tissue of origin. Source data

The MTBP matches the cancer biomarkers aggregated across these knowledge bases with the variants observed in the tumor for (i) a specific nucleotide and/or protein amino acid change (e.g., *BRCA1*:c.5468-1G>A or *KIT*:p.D572A); (ii) a variant category (*e.g., EGFR* in-frame deletions in exon 19); or (iii) a functional entity (e.g., *FLT3* oncogenic mutations, as guided by the MTBP functional interpretation) (Fig. 4a). However, as mentioned before, variant actionability must also take into account tumor-context considerations beyond the mere variant match, such as the concordance between the biomarker and patient’s cancer type (or a subtype thereof), the presence of co-occurring alterations that can influence the biomarker effect and the level of evidence that supports its clinical utility at present (Fig. 4a). The MTBP pipeline factors in these considerations as appropriate and reports the results following the European Society for Medical Oncology (ESMO) Clinical Actionability of Molecular Targets (ESCAT) scale^22^, which is an extension of that previously presented by American expert associations^23^ (Fig. 4b).

**Fig. 5:**
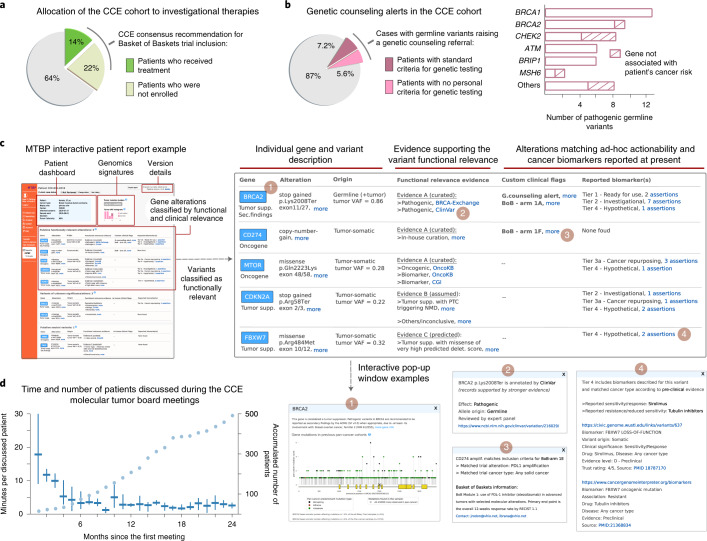
Use of the MTBP in Cancer Core Europe. **a**, Patients of the CCE cohort recommended for one of the Basket of Baskets trial arms available at the moment of the molecular tumor board discussion. The pie chart shows the proportion of these patients that were not enrolled due to clinical deterioration and/or screening failures. For the remaining cases, other investigational treatments were evaluated in the respective patient’s medical institution without requiring CCE consensus (data not shown). **b**, Patients in which the MTBP issued an alert due to germline variant(s) associated with inherited cancer risk (restricted to the 375 patients with sequencing of both tumor and normal-paired samples). The pie chart shows the proportion of these cases that would have been overlooked by current germline testing guidelines, as variant carriers did not meet personal criteria. Bar plots detail the genes in which these pathogenic variants were identified and whether they are currently described as increasing the risk for the respective patient’s index cancer type. **c**, The MTBP reports used in CCE contain an integrated dashboard with clinical and pathology information, details of the sequencing assay(s) and a version control of the resources used to annotate the data. Clinically relevant molecular signatures are listed together with individual gene alterations, the latter organized in three different tables according to their functional classification (functionally relevant, unclassified and functionally neutral). These tables summarize the evidence supporting the variants’ functional classification and their associated actionability, including in-house clinical initiatives (prioritizing eligibility for clinical trials available across the CCE network) and matching with cancer biomarkers reported to date (diagnosis, prognosis and drug response, tiered according to the ESMO-ESCAT scale; see Fig. 4). Access to detailed information is provided by the use of interactive elements in the HTML report, as exemplified here by the pop-up windows opened in 1–4. **d**, The lines represent the time (median and IQR) devoted to discussing each patient’s case during the CCE virtual molecular tumor board meetings. The circles indicate the accumulated number of reviewed cases (overall 500 patients). BoB, Basket of Baskets; VAF, variant allele frequency. Source data

The highest level of actionability corresponds to genomic alterations matching on-label prescriptions or clinical expert group recommendations, and thus ready to be used in routine clinical practice (ESCAT level I; Fig. 4c). However, in the context of CCE initiatives, we mostly profile tumors of patients without standard-of-care therapeutic options available. Consequently, investigational and off-label drug opportunities based on preliminary clinical (ESCAT levels II and III) or even preclinical (ESCAT level IV) evidence are also considered. In these cases, we prioritize the allocation of patients to clinical trials, and one key feature of the MTBP is therefore to detect cases eligible for those open for recruitment across the connected centers, as detailed next. Of note, the CCE network hosts the Basket of Baskets study, a European multiarm phase 2 basket trial for advanced tumors selected according to predefined molecular profiles (NCT03767075). Overall, 36% of the patients of the CCE cohort presented here were recommended for one of the Basket of Baskets treatment arms available at the moment of the molecular tumor board discussion (Fig. 5a). These were mostly associated with the use of immune checkpoint inhibitors in the presence of loss-of-function events in DNA damage repair genes, as estimated by the MTBP variant interpretation (Methods). However, the majority (60%) of these patients were not finally enrolled in the Basket of Baskets trial, mostly because of the deterioration of their clinical condition or subsequent screening failures. This further emphasizes the importance of deploying systems that can support an efficient and rapid trial recruitment at the point of clinical decision-making.

The MTBP retrieves the trials’ eligibility criteria from in-house databases gathering clinical, pathological and molecular requisites. This information is curated following an ad-hoc syntax adapted to the growing complexity of cancer biomarkers, which are defined by the presence (or absence) of a given combination of genomic alterations and/or genomic signatures. This syntax also defines prioritization rules in case that the allocation to multiple trials is possible, as well as variant interpretation nuances to be used by the MTBP interpretation framework. One example of the latter is the evidence required for considering variants in a given actionable gene as functionally relevant; in general, we only match clinical trials with tumor variants whose effect is based on well-curated studies or bona fide biological assumptions, but lower evidence, such as bioinformatic predictions, is also considered for emerging biomarkers associated with less characterized genes. Upfront agreement on these details enables the MTBP to refine the actionability flags issued in the reports, which facilitates efficient discussions during the molecular tumor board meetings and increases the consistency of the clinical decision-making.

Importantly, the MTBP can be used to automate the detection of other events of potential clinical relevance. For example, and in collaboration with the CCE genetic counseling task force, we have established unified criteria to flag germline variants requiring genetic specialist referral (Methods). As a result, the MTBP issued genetic counseling alerts for 49 germline variants in 48 individuals (57 ± 13 years) of the CCE cohort, which represent 13% of those with paired tumor/normal samples sequencing available (with a cancer type distribution similar to that of the overall cohort; Table 1). Incidentally, three (6%) of these variants showed a low variant allele frequency in the tumor sample, and thus, they would not have been contemplated as of potential germline origin with tumor-only sequencing data, as per published criteria^24^. Genetic counselor review deemed these findings as of clinical relevance in all the cases, although close to half (44%) of the variant carriers did not meet personal criteria for clinical germline testing^25,26^ (Fig. 5b). Moreover, a considerable (18%) proportion of these pathogenic germline variants were found in genes not associated with the patient’s index cancer, which further complicates their discovery via standard guidelines-directed genetic testing. These results illustrate the importance of the oncology setting for screening hereditary cancer susceptibility variants^27^ and the value of the MTBP to streamline that task.

### The MTBP technology

The MTBP provides a single unified framework for sharing and harnessing NGS data across CCE centers. Deidentified patient clinical and pathological information is fetched from a centralized electronic case report forms system, whereas sequencing data files are retrieved from different institutional and external laboratories. Data transfer, storage and access are implemented by a set of technical measures in accordance with the European legal framework under compliance with data protection regulation (Methods). After data capture, the system triggers a number of pipelines for data integrity control, format harmonization and variant interpretation as appropriate. This ultimately creates the corresponding patient reports, which are immediately shared with the clinical investigators. The whole process is fully automated and thus performed in a negligible amount of time, which dramatically reduces the efforts required for case preparation. This facilitates our current turnaround time, which is less than 14 days from biopsy collection to report generation, as required in patients whose clinical condition can rapidly deteriorate^28^.

The MTBP patient reports are HTML web-based documents accessible for the clinical investigators via a secure online platform^2^. These reports are discussed in weekly virtual molecular tumor board meetings, in which members of each CCE center connect from different locations and agree on clinical recommendations. As discussed before, genomic alterations are flagged in the MTBP report according to predefined expert actionability criteria, and all the results appear systematically organized in a user-friendly, readily interpretable view (Fig. 5c). In addition, further information and variant annotation details are accessible via interactive elements of the HTML report, which empowers an in-depth revision of the content and supporting evidence. Although the MTBP can also distribute simplified reports in PDF format, we believe that working with interactive data-rich documents is more appropriate in the context of academic medical centers, in which molecular tumor boards discuss complex cases and serve as a venue for continued education in genomics-driven oncology. Of note, we observed a learning curve to use the MTBP system lasting for approximately 25 patients (Fig. 5d). After that, the amount of time devoted to discussing each case averaged less than three minutes, which is key for scaling the process to a large number of patients.

**Extended Data Fig. 1:**
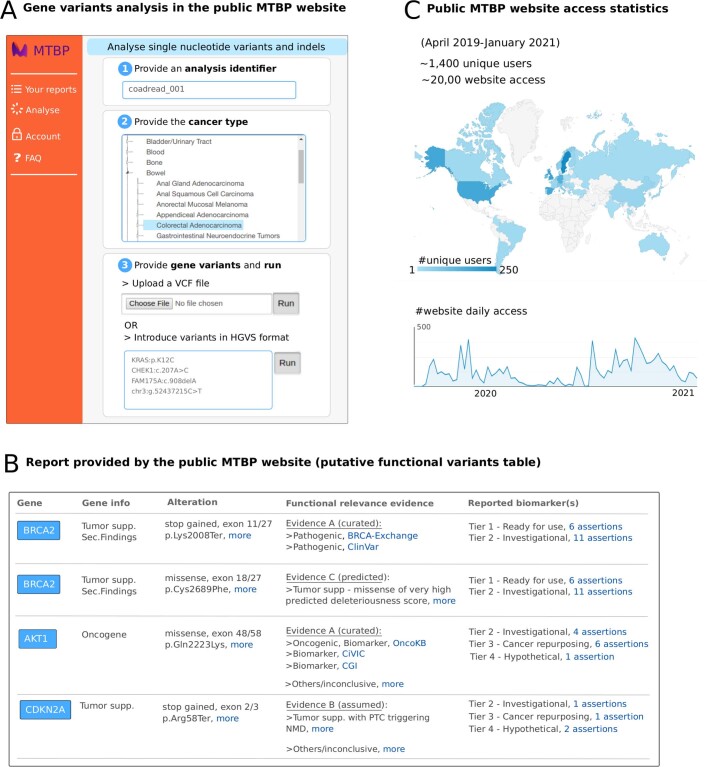
Public version of the Molecular Tumor Board Portal. **a**, The public MTBP is an open resource for research purposes that provides a lightweight version of the MTBP analytical pipeline. This public resource is accessed via a website (https://www.mtbp.org/) that offers an interface to upload the gene variants to analyze. At the moment of writing this manuscript, the public MTBP only supports the analysis of single-nucleotide variants and small indels. **b**, The public version of the MTBP does not issue actionability flags that require information that cannot be inferred from the generic input employed here and/or require interpretation nuances that may differ across investigators/institutions (such as matching with a given portfolio of clinical trials or the identification of germline incidental findings). Instead, the public MTBP provides a general interpretation of the functional and predictive relevance of the uploaded variants, with the aim of supporting a detailed review of the user according to his/her specific needs. **c**, Public MTBP website activity from the date of its release until the moment of writing this manuscript.

At the moment of writing, the MTBP system used by CCE initiatives supports the interpretation of genomics data (mutations, copy-number alterations, structural variants and mutational signatures) and has recently incorporated gene expression analysis. In addition, and in the context of ongoing efforts to implement new clinical trial designs, we are currently working on the incorporation of emerging tumor profiling technologies such as proteomics, ex vivo drug screening and digital pathology. Ultimately, we envision the MTBP as a catalyst for systems-based precision oncology, capable of integrating multiple levels of molecular and imaging data and inform treatment decisions throughout the patient’s disease course. In addition, we have also created an open website (https://mtbp.org) that provides access to a lightweight version of the MTBP analytical framework. This public resource is intended for research purposes and only supports a general interpretation of gene variants that may be of interest for investigators outside our network (Extended Data Fig. 1).

## Discussion

The MTBP provides a unified platform for implementing precision oncology strategies in a truly collaborative manner. As the complexity of cancer biomarkers continues to grow, automating the interpretation and reporting of sequencing results decreases the need for manual procedures and facilitates rapid, comprehensive and consistent clinical decision-making. In addition, the MTBP creates the infrastructure to systematically gather the molecular and clinical information in a ‘biorepository’ of data, which supports the discovery of new biomarkers and insights for future trial designs. However, deploying the MTBP across the CCE network raised multiple challenges, such as (i) ensuring the interoperability with the information technology systems of each connected center; (ii) automating the retrieval of clinical, pathological and sequencing data provided by different facilities; (iii) developing user-friendly interfaces for distinct user types, such as medical practitioners, project managers and data analysts; (iv) coordinating the efforts to agree on variant interpretation criteria and actionability prioritization; and (v) creating the associated resources, such as a database with up-to-date information of the clinical trials open for recruitment across the network. These tasks require expertise from domains such as medical software regulation, cybersecurity and front-end development, which is not easily available in the academic setting and thus creates needs for collaboration with industry partners. In conclusion, we believe that streamlining digital systems like the MTBP at the point of care is key to better address the challenges of delivering biomarker-driven oncology at scale, but the success of these initiatives relies on the long-term investment needed to develop and maintain the technology.

## Methods

### Ethical regulation

The Vall d’Hebron Institute of Oncology is the sponsor of the Basket of Baskets trial. The protocol was submitted through the Voluntary Harmonization Procedure and approved by the Medicines & Healthcare products Regulatory Agency in the United Kingdom. Subsequently, the competent authorities in Spain (Agencia Española de Medicamentos y Productos Sanitarios), France (Agence nationale de sécurité du medicament et des produits de santé), Germany (Bundesinstitut für Impfstoffe und biomedizinische Arzneimittel), the Netherlands (Centrale Commissie Mensgebonden Onderzoek) and Sweden (Läkemedelsverket) provided local approval. Ethics committee approvals have been obtained in Spain, the United Kingdom, France, the Netherlands and Sweden under EduraCT project number 2018-005108-89. All patients signed an informed consent form for preregistration and another for the trial in the case of recruitment. The clinical and sequencing data transfer, storage and access complies with European legal and ethical regulations as appropriate.

### Statistics and reproducibility

This article includes a description of a consecutive cohort of advanced tumor patients preregistered in the Basket of Baskets (NCT03767075) trial during a 2-yr period. No statistical method was used to predetermine sample size. No data were excluded from the analyses. The experiments were not randomized. The investigators were not blinded to allocation during experiments and outcome assessment. The article describes general tumor genomic findings aggregated at the cohort level, and the resulting descriptive numbers (no statistic tests were necessary) are available in the text, tables and figures.

### MTBP in CCE (CCE-MTBP)

Patient clinical and tumor pathological information is gathered in pseudo-anonymized electronic case report forms (ALEA system; https://www.aleaclinical.eu/). Molecular assay results are provided by CCE institutional facilities and commercial laboratories. Data is automatically transferred to the CCE-MTBP through secure protocols as appropriate. Upon receival of each data file the CCE-MTBP checks the integrity of the content and (if no issues are detected) several analytical pipelines are triggered to process the results and create the corresponding patient-centric report, which is made available to clinical investigators via an online secure platform. CCE-MTBP data governance is addressed through multiple technical, physical and legal measures (not disclosed here). The logs of each CCE-MTBP pipeline run are manually reviewed by the MTBP developers as part of our standard operating procedures, and email alerts are sent as appropriate when new results are available.

### Public instance of the MTBP (public-MTBP)

Access to a lightweight version of the MTBP genomics analytical pipeline has been made freely available at https://mtbp.org. At the moment of writing, the public-MTBP supports the analysis of single-nucleotide variants and small indels, which can be manually uploaded via a VCF file (hg19/GRCh37 and hg38/GRCh38 coordinates supported) or through a free text box for genomic, cDNA and/or protein-based mutations (HGVS syntax with several reference systems supported). The public-MTBP provides a general interpretation of the functional and predictive relevance of the uploaded variants but does not issue actionability flags (such as potential eligibility for clinical trials or genetic counseling alerts) that require additional patient information. Instead, the public-MTBP aims for a comprehensive variant annotation to support a detailed review according to different user’s needs.

### Genomic alterations considered for the Basket of Baskets study

Genomic alterations considered for the use of atezolizumab in the first treatment module opened for the Basket of Baskets (NCT03767075) study opened during the evaluation of the CCE patient cohort described here are the following: BRCA1 or BRCA2 loss-of-function mutations (arm 1A); MLH1, MSH2, MSH6 or PMS2 loss-of-function mutations (arm 1B); POLE or POLD1 switch-of-function mutations (arm 1C); intermediate or high tumor mutation burden without an apparent mechanism for DNA damage repair malfunction (arm 1D); loss of function observed in other DNA damage repair genes (arm 1E); and CD274 (PDL-1) copy-number amplifications (arm 1F). Note that the Basket of Baskets is a dynamic platform trial in which new treatment modules are progressively opened or closed through the corresponding amendments.

### List of genes evaluated for germline variants conferring inherited increased cancer risk

These are the genes recommended by the American College of Medical Genetics and Genomics (ACMG SF v2.0 update), plus some additions (marked with an asterisk) agreed to be clinically actionable by the CCE Genetic Counseling Task Force. Of note, the MTBP issues genetic counseling alerts only if the germline variants observed in these genes are estimated to be loss of function based on well-curated evidence and/or bona fide biological assumptions, regardless of patient clinical information: APC, ATM*, BMPR1A, BRCA1, BRCA2, BRIP1*, CDH1*, CHEK2, MEN1, MLH1, MSH2, MSH6, MUTYH (only reported for homozygous or compound heterozygous MUTYH mutations), NF2, PALB2*, PMS2, PTEN, RAD51C*, RAD51D*, RB1, RET, SDHB, SDHC, SDHD, SMAD3, SMAD4, STK11, TGFBR1, TGFBR2, TP53, TSC1, TSC2, VHL and WT1.

### MTBP annotation

The MTBP uses a combination of in-house and publicly available^17,18^ bioinformatic tools to transform the different formats produced by the CCE-MTBP data providers and the different variant nomenclatures accepted by the public-MTBP into a single unambiguous representation system. The MTBP annotates these data with multiple resources based on the classification schemes shown in Fig. 2 (functional relevance) and Fig. 4 (predictive relevance). Among these resources, the MTBP uses third-party knowledge bases that (i) formalize information about the variants’ effect by using predefined processes, (ii) are based on published biomedical literature and (iii) are committed to periodical updates. Variant nomenclature, terminology and taxonomy systems used to annotate the data are also unified across knowledge bases with a semiautomatic MTBP pipeline that is regularly updated. Additional filtering distinguishes knowledge base assertions supported by weaker or inconclusive evidence, as extracted from the corresponding metadata as appropriate (e.g., less than two or three stars in the ClinVar or CIViC evidence rating, respectively). Only resources open for academic research purposes are used in the public-MTBP^4–10^. The MTBP also implements bona fide biological assumptions to estimate the relevance of variants without conclusive curated effects, which are largely based on established rules to identify loss-of-function variants in Mendelian genes^11^ and subsequent refinements^12^. In the case of tumor suppressors (see Fig. 2d), these include canonical splice site disruptions and variants leading to a premature stop codon (likely) triggering nonsense-mediated decay mechanisms, which are considered very strong criteria (so-called PVS1); variants truncating/disrupting protein regions that are crucial for the tumor suppressor function, which are considered strong supporting criteria (PVS1_Strong) (of note, the identification of these essential protein regions is refined by analyzing location and consequence type of known loss-of-function variants, as gathered from the knowledge bases aggregated by the MTBP (manuscript in preparation)); and variants truncating/disrupting more than 10% of the wild-type tumor suppressor protein, which are considered strong supporting criteria (PVS1_Strong). Finally, if none of the previous evidence is conclusive, then the MTBP evaluates variants’ relevance with computational methods selected according to our own benchmarking (next section). On the other hand, the MTBP factors in additional tumor-context considerations to classify the actionability of variants deemed as functionally relevant following the ESMO-ESCAT recommendations^22^. Of note, the MTBP introduces two minor modifications to this classification framework (see Fig. 4b) due to (i) the lack of structured information in the knowledge bases to infer the trial design details and (ii) the use of biomarkers reported by clinical observations but only supported by case reports.

### Data analysis

Knowledge bases that were used to obtain the results shown here are ClinVar, BRCA Exchange, CIViC, OncoKB, Cancer Genome Interpreter, 1000 Genomes and gnomAD^4–10^. Mutation hotspots were considered separately for missense variants and in-frame insertions/deletions using *P* values lower than 5% as statistically significant following the methods based on the two- and three-dimensional protein structures, respectively^14,15^. Benchmarking of functional impact score methods was performed using the aggregated content of the aforementioned knowledge bases. Importantly, thresholds were also selected based on this benchmarking (Fig. 2e); in detail, missense variants with very high (>30) and very low (<10) Phred combined annotation dependent depletion^16^ scores exhibited 90% of true functional and neutral calls, respectively, as required for being considered strong criteria^3^. In addition, their stringency was observed by simulating in silico all the possible nucleotide changes that can lead to missense variants in the most recurrently mutated tumor suppressor genes (Fig. 2e), which showed that in most cases, the variants remained unclassified when using these thresholds. All types of evidence supporting the functional relevance of the observed tumor variants were used to match them with cancer biomarkers in the figures, unless stated otherwise. The duration for discussing a patient’s case in the CCE molecular tumor board meetings (Fig. 5d) encompassed the presentation of the clinical summary, the discussion of the CCE-MTBP report content and the decision-voting process to reach a clinical recommendation.

### Reporting Summary

Further information on research design is available in the Nature Research Reporting Summary linked to this article.

## Supplementary information

Supplementary Information Full list of CCE consortium members.

Reporting Summary

## Data Availability

Sequencing data have been deposited to the European Genome–phenome Archive, which is hosted by the European Bioinformatics Institute and the Centre for Genomic Regulation. Because of patient privacy constraints, the data are under controlled access and available upon reasonable request to the CCE Basket of Baskets Data Access Committee (bob@vhio.net) based on European Genome–phenome Archive terms (see accession number EGAS00001005893 for further details). Source data are provided with this paper.

## Source data

Source Data Fig. 1 Numerical source data.
Source Data Fig. 2 Numerical source data.
Source Data Fig. 3 Numerical source data.
Source Data Fig. 4 Numerical source data.
Source Data Fig. 5 Numerical source data.

## Code availability

Aggregated results shown in this article were created using Python (>3.0) NumPy, Pandas and Matplotlib open-source libraries. The code for the MTBP components reproducing the variant annotation framework discussed in the manuscript has been deposited to Zenodo with access restricted for research use only (see 10.5281/zenodo.6376428 for terms of use details). Access to other components available in MTBP production systems can be provided upon agreement with the copyright holders.
