# Author Correction: The Molecular Tumor Board Portal supports clinical decisions and automated reporting for precision oncology

**DOI:** 10.1038/s43018-022-00378-x

**Published:** 2022-04-21

**Authors:** David Tamborero, Rodrigo Dienstmann, Maan Haj Rachid, Jorrit Boekel, Adria Lopez-Fernandez, Markus Jonsson, Ali Razzak, Irene Braña, Luigi De Petris, Jeffrey Yachnin, Richard D. Baird, Yohann Loriot, Christophe Massard, Patricia Martin-Romano, Frans Opdam, Richard F. Schlenk, Claudio Vernieri, Michele Masucci, Xenia Villalobos, Elena Chavarria, Cancer Core Europe consortium, Shubha Anand, Danny Baars, Svetlana Bajalica-Lagercrantz, Richard Baird, Mariska Bierkens, Lennart Blomqvist, Costanza Bono, Luigi De Petris, Gary J. Doherty, Arnauld Forest, Valentina Fornerone, Paola Gabaldi, Felix Haglund, Johan Hartman, Peter Horak, Tanja Jutzi, Mary Kasanicki, Simon Kreutzfeldt, Lucian Le Cornet, Rolf Lewensohn, Johan Lindberg, Carlos Lopez, Andreas Lundqvist, Jose-Ezequiel Martin, Gerrit Meijer, Susana Muñoz, Maud Ngo Camus, Claudio Nicotra, Paolo Nuciforo, Petra Oberrauch, Päivi Östling, Laura Pelz, Alejandro Piris-Gimenez, Elena Provenzano, Etienne Rouleau, John Rowell, Omar Saavedra, Giovanni Scoazec, Kenneth Seamon, Marc Tischkowitz, Lizet van der Kolk, Ruud van der Noll, Maria Vieito, Daniel Vis, Ana Vivancos, Christina von Gertten, Anders Wennborg, Lodewyk Wessels, Valtteri Wirta, Judith Balmaña, Giovanni Apolone, Carlos Caldas, Jonas Bergh, Ingemar Ernberg, Stefan Fröhling, Elena Garralda, Claes Karlsson, Josep Tabernero, Emile Voest, Jordi Rodon, Janne Lehtiö

**Affiliations:** 1 Department of Oncology and Pathology, Karolinska Institutet, Science for Life Laboratory, Stockholm, Sweden; 2 Medical Oncology, Oncology Data Science, Vall d’Hebron Institute of Oncology (VHIO), Barcelona, Spain; 3 Hereditary Cancer Genetics Group, Vall d’Hebron Institute of Oncology (VHIO), Barcelona, Spain; 4 Medical Oncology Department, Vall d’Hebron University Hospital, Vall d’Hebron Institute of Oncology (VHIO), Barcelona, Spain; 5 Department of Oncology and Pathology, Karolinska Institutet, Theme Cancer, Karolinska Comprehensive Cancer Center, Karolinska University Hospital, Stockholm, Sweden; 6 Cancer Research UK Cambridge Centre, Cambridge, UK; 7 Département d’Innovation Thérapeutique et d’Essais Précoces, Gustave Roussy, Université Paris-Saclay, Villejuif, France; 8 The Netherlands Cancer Institute, Amsterdam, the Netherlands; 9 NCT Trial Center, German Cancer Research Center, Heidelberg University Hospital, Heidelberg, Germany; 10 Fondazione IRCCS Istituto Nazionale dei Tumori, Milan, Italy; 11 IFOM, FIRC Institute of Molecular Oncology, Milan, Italy; 12 Department of Microbiology, Tumor and Cell Biology, Karolinska Institutet, Stockholm, Sweden; 13 Vall d’Hebron Institute of Oncology (VHIO), Barcelona, Spain; 14 Scientific Directorate, Fondazione IRCCS Istituto Nazionale dei Tumori, Milan, Italy; 15 National Center for Tumor Diseases Heidelberg, German Cancer Research Center, Heidelberg, Germany; 16 Department of Oncology and Pathology, Karolinska Institutet, Stockholm, Sweden; 17 Department of Hematology, Karolinska University Hospital, Stockholm, Sweden; 18 Oncode Institute, Utrecht, the Netherlands; 19 Department of Investigational Cancer Therapeutics, University of Texas MD Anderson Cancer Center, Houston, TX USA; 20 Clinical Proteomics Unit, Department of Oncology and Pathology, Karolinska Institutet, Science for Life Laboratory, Karolinska University Hospital, Stockholm, Sweden; 21 Cancer Molecular Diagnostics Laboratory, Department of Oncology, University of Cambridge, Cambridge, UK; 22 Department of Scientific Administration, The Netherlands Cancer Institute, Amsterdam, The Netherlands; 23 Department of Oncology-Pathology, Karolinska Institutet, Stockholm, Sweden; 24 Department of Clinical Genetics, Karolinska University Hospital, Stockholm, Sweden; 25 Department of Pathology, The Netherlands Cancer Institute, Amsterdam, The Netherlands; 26 Department of Imaging and Physiology, Karolinska University Hospital, Stockholm, Sweden; 27 Department of Molecular Medicine and Surgery, Karolinska Institutet, Stockholm, Sweden; 28 Department of Oncology, Cambridge University Hospitals NHS Foundation Trust, Cambridge Biomedical Campus, Cambridge, UK; 29 Institut Gustave Roussy, Villejuif, France; 30 Department of Oncology-Pathology, Karolinska Institutet, Stockholm, Sweden; 31 Clinical Pathology and Cancer Diagnostics, Karolinska University Hospital, Stockholm, Sweden; 32 Department of Oncology-Pathology, Karolinska Institutet, Stockholm, Sweden; 33 Division of Translational Medical Oncology, National Center for Tumor Diseases Heidelberg and German Cancer Research Center, Heidelberg, Germany; 34 National Institute for Health Research Cambridge Biomedical Research Centre, University of Cambridge, Cambridge, UK; 35 NCT Trial Center, German Cancer Research Center and Heidelberg University Hospital, Heidelberg, Germany; 36 Theme Cancer, Karolinska Comprehensive Cancer Center, Stockholm, Sweden; 37 Department of Oncology-Pathology, Karolinska Institutet, Stockholm, Sweden; 38 Department of Medical Epidemiology and Biostatistics, Science for Life Laboratory, Karolinska Institutet, Stockholm, Sweden; 39 Business Development Area, Vall d’Hebron Institute of Oncology (VHIO), Vall d’Hebron Barcelona Hospital Campus, Barcelona, Spain; 40 Clinical Research Support Unit, Vall d’Hebron Institute of Oncology (VHIO), Vall d’Hebron Barcelona Hospital Campus, Barcelona, Spain; 41 DITEP– Drug Development Department, Institut Gustave Roussy, Villejuif, France; 42 Molecular Oncology Group, Vall d’Hebron Institute of Oncology (VHIO), Vall d’Hebron Barcelona Hospital Campus, Barcelona, Spain; 43 Department of Oncology and Pathology, Science for Life Laboratory, Karolinska Institutet, Stockholm, Sweden; 44 Research Coordination Area, Vall d’Hebron Institute of Oncology (VHIO), Vall d’Hebron Barcelona Hospital Campus, Barcelona, Spain; 45 Tumor Genetic Lab, Institut Gustave Roussy, INSERM UMR 981 Villejuif, France; 46 Cancer Core Europe, Institut Gustave Roussy, Villejuif, France; 47 Medical Oncology Department, Vall d’Hebron Institute of Oncology (VHIO), Hospital Universitari Vall d’Hebron, Vall d’Hebron Barcelona Hospital Campus, Barcelona, Spain; 48 Family Cancer Clinic, The Netherlands Cancer Institute, Amsterdam, the Netherlands; 49 Drug development Unit, Vall d’Hebron Institute of Oncology (VHIO), Barcelona, Spain; 50 Oncode Institute, The Netherlands Cancer Institute, Amsterdam, The Netherlands; 51 Cancer Genomics Group, Vall d’Hebron Institute of Oncology (VHIO), Vall d’Hebron Barcelona Hospital Campus, Barcelona, Spain; 52 Department of Microbiology, Tumor and Cell Biology, Science for Life Laboratory, Karolinska Institutet, Stockholm, Sweden

**Keywords:** Cancer genomics, Cancer genetics, Cancer, Computational biology and bioinformatics

Correction to: *Nature Cancer* 10.1038/s43018-022-00332-x, published online 24 February 2022.

In this article, the Code availability section has been updated to clarify conditions for access and to revise the repository link. The new statement now reads “Aggregated results shown in this article were created using Python (>3.5) NumPy, Pandas and Matplotlib open-source libraries. The code for the MTBP components reproducing the variant annotation framework discussed in the manuscript has been deposited to Zenodo with access restricted for research use only (see 10.5281/zenodo.6376428 for terms of use details). Access to other components available in MTBP production systems can be provided upon agreement with the copyright holders.” The change has been made in the HTML and PDF versions of the article.

