# Supplementary material for: The Molecular Tumor Board Portal supports clinical decisions and automated reporting for precision oncology
Source: Nat Cancer. 2022 Feb 24;3(2):251–61. doi: 10.1038/s43018-022-00332-x (PMC8882467; doi:10.1038/s43018-022-00332-x)
Supplement: Supplementary file 1 — Full list of CCE consortium members. [file 43018_2022_332_MOESM1_ESM.pdf]

nature portfolio

https://doi.org/10.1038/s43018-022-00332-x

Supplementary information

The Molecular Tumor Board Portal supports clinical decisions and automated reporting for precision oncology

In the format provided by the authors and unedited

Cancer Core Europe Consortium

Shubha Anand(21), Giovanni Apolone(14), Danny Baars(22), Svetlana Bajalica-Lagercrantz(23), Richard Baird(6), Judith Balmaña(3), Jonas Bergh(5), Mariska Bierkens(24), Lennart Blomqvist(25), Jorrit Boekel(1), Costanza Bono(14), Irene Braña(4), Carlos Caldas(6), Elena Chavarria(13), Luigi De Petris(5), Rodrigo Dienstmann(2), Gary J. Doherty(26), Ingemar Ernberg(12), Arnauld Forest(27), Valentina Fornerone(14), Stefan Fröhling(15), Paola Gabaldi(14), Elena Garralda(4), Felix Haglund(28), Johan Hartman(29), Peter Horak(30), Markus Jonsson(1), Tanja Jutzi(30), Claes Karlsson(16)(17), Mary Kasanicki(31), Simon Kreutzfeldt(30), Lucian Le Cornet,(32) Janne Lehtiö(20), Rolf Lewensohn(33), Johan Lindberg(34), Carlos Lopez(35), Adria Lopez-Fernandez(3), Yohann Loriot(7), Andreas Lundqvist(29), Jose-Ezequiel Martin (21), Patricia Martin-Romano(7), Christophe Massard(7), Michele Masucci(12), Gerrit Meijer(24), Susana Muñoz(36), Maud Ngo Camus(37), Claudio Nicotra(37), Paolo Nuciforo(38), Petra Oberrauch(30), Frans Opdam(8), Päivi Östling(39), Laura Pelz(30), Alejandro Piris-Gimenez(40), Elena Provenzano(31), Maan Haj Rachid(1), Ali Razzak(1), Jordi Rodon(4)(19), Etienne Rouleau(41), John Rowell(42), Omar Saavedra(43), Richard F. Schlenk(9), Giovanni Scoazec(14), Kenneth Seamon(6), Josep Tabernero(4), David Tamborero(1), Marc Tischkowitz(31), Lizet van der Kolk(44), Ruud van der Noll(22), Claudio Vernieri(10)(11), Maria Vieito(45), Xenia Villalobos(13), Daniel Vis(46), Ana Vivancos(47), Emile Voest(8)(18), Christina von Gertten(29), Anders Wennborg(12), Lodewyk Wessels(46), Valtteri Wirta(48), Jeffrey Yachnin(5)

Affiliations:

(1) Dep. of Oncology and Pathology, Karolinska Institutet, Science for Life Laboratory, Stockholm, Sweden

(2) Medical Oncology - Oncology Data Science, Vall d'Hebron Institute of Oncology (VHIO), Barcelona, Spain

(3) Hereditary Cancer Genetics Group. Vall d'Hebron Institute of Oncology (VHIO), Barcelona, Spain

(4) Medical Oncology Department, Vall d'Hebron University Hospital and Institute of Oncology (VHIO), Barcelona, Spain.

(5) Dep. of Oncology and Pathology, Karolinska Institutet, Theme Cancer, Karolinska Comprehensive Cancer Center, Karolinska University Hospital, Stockholm, Sweden

(6) Cancer Research UK Cambridge Centre, Cambridge, UK

(7) Département d'Innovation Thérapeutique et d'Essais Précoces, Gustave Roussy, Université Paris-Saclay, Villejuif, France
(8) The Netherlands Cancer Institute, Amsterdam, the Netherlands
(9) NCT Trial Center, German Cancer Research Center (DKFZ) and Heidelberg University Hospital, Heidelberg, Germany
(10) Fondazione IRCCS Istituto Nazionale dei Tumori, Milan, Italy
(11) IFOM, the FIRC Institute of Molecular Oncology, Milan, Italy
(12) Department of Microbiology, Tumor and Cell Biology, Karolinska Institutet, Stockholm, Sweden
(13) Vall d'Hebron Institute of Oncology (VHIO), Barcelona, Spain
(14) Fondazione IRCCS Istituto Nazionale dei Tumori, Scientific Directorate, Milan, Italy
(15) National Center for Tumor Diseases (NCT) Heidelberg, German Cancer Research Center (DKFZ), Heidelberg, Germany
(16) Department of Oncology-Pathology, Karolinska Institutet, Stockholm, Sweden
(17) Department of Hematology, Karolinska University Hospital, Stockholm, Sweden
(18) Oncode Institute
(19) Department of Investigational Cancer Therapeutics, U.T.M.D. Anderson Cancer Center, Houston, Texas
(20) Dep. of Oncology and Pathology, Karolinska Institutet, SciLifeLab, Clinical Proteomics Unit, Karolinska University Hospital, Stockholm, Sweden
(21) Cancer Molecular Diagnostics Laboratory, Department of Oncology, University of Cambridge, Cambridge, UK.
(22) Department of Scientific Administration, The Netherlands Cancer Institute, Amsterdam, The Netherlands.
(23) Department of Oncology-Pathology, Karolinska Institutet, Stockholm, Sweden. Department of Clinical Genetics, Karolinska University Hospital, Stockholm, Sweden.
(24) Department of Pathology, The Netherlands Cancer Institute, Amsterdam, The Netherlands.
(25) Department of Imaging and Physiology, Karolinska University Hospital, Stockholm, Sweden. Department of Molecular Medicine and Surgery, Karolinska Institutet, Stockholm, Sweden.
(26) Department of Oncology, Cambridge University Hospitals NHS Foundation Trust, Cambridge Biomedical Campus, Cambridge, UK.
(27) Institut Gustave Roussy, Villejuif, France.
(28) Department of Oncology-Pathology, Karolinska Institutet, Stockholm, Sweden. Clinical Pathology and Cancer Diagnostics, Karolinska University Hospital, Stockholm, Sweden.

(29) Department of Oncology-Pathology, Karolinska Institutet, Stockholm, Sweden.
(30) Division of Translational Medical Oncology, National Center for Tumor Diseases Heidelberg and German Cancer Research Center, Heidelberg, Germany.
(31) National Institute for Health Research Cambridge Biomedical Research Centre, University of Cambridge, Cambridge, UK.
(32) NCT Trial Center, German Cancer Research Center and Heidelberg University Hospital, Heidelberg, Germany.
(33) Theme Cancer, Karolinska Comprehensive Cancer Center, Stockholm, Sweden. Department of Oncology-Pathology, Karolinska Institutet, Stockholm, Sweden.
(34) Department of Medical Epidemiology and Biostatistics, Science for Life Laboratory, Karolinska Institutet, Stockholm, Sweden.
(35) Business Development Area, Vall d’Hebron Institute of Oncology, Vall d’Hebron Barcelona Hospital Campus, Barcelona, Spain.
(36) Clinical Research Support Unit, Vall d’Hebron Institute of Oncology, Vall d’Hebron Barcelona Hospital Campus, Barcelona, Spain.
(37) DITEP– Drug Development Department, Institut Gustave Roussy, Villejuif, France.
(38) Molecular Oncology Group, Vall d’Hebron Institute of Oncology, Vall d’Hebron Barcelona Hospital Campus, Barcelona, Spain.
(39) Department of Oncology and Pathology, Science for Life Laboratory, Karolinska Institutet,Stockholm, Sweden.
(40) Research Coordination Area, Vall d’Hebron Institute of Oncology, Vall d’Hebron Barcelona Hospital Campus, Barcelona, Spain.
(41) Tumor Genetic Lab, Institut Gustave Roussy, INSERM UMR 981, Villejuif, France.
(42) Cancer Core Europe, Institut Gustave Roussy, Villejuif, France.
(43) Medical Oncology Department, Vall d’Hebron Institute of Oncology, Hospital Universitari Vall d’Hebron, Vall d’Hebron Barcelona Hospital Campus,Barcelona, Spain.
(44) Family Cancer Clinic, The Netherlands Cancer Institute, Amsterdam, the Netherlands.
(45) Drug development Unit, Vall d'Hebron Institute of Oncology, Barcelona, Spain.
(46) Oncode Institute, The Netherlands Cancer Institute, Amsterdam, The Netherlands.
(47) Cancer Genomics Group, Vall d’Hebron Institute of Oncology, Vall d’Hebron Barcelona Hospital Campus, Barcelona, Spain.
(48) Department of Microbiology, Tumor and Cell Biology, Science for Life Laboratory, Karolinska Institutet, Stockholm, Sweden.
