# Supplementary material for: The Molecular Tumor Board Portal supports clinical decisions and automated reporting for precision oncology
Source: Nat Cancer. 2022 Feb 24;3(2):251–61. doi: 10.1038/s43018-022-00332-x (PMC8882467; doi:10.1038/s43018-022-00332-x)
Supplement: Supplementary file 2 — Reporting Summary [file 43018_2022_332_MOESM2_ESM.pdf]

nature research

Corresponding author(s): David Tamborero

Last updated by author(s): Dec 21, 2021

Reporting Summary

Nature Research wishes to improve the reproducibility of the work that we publish. This form provides structure for consistency and transparency in reporting. For further information on Nature Research policies, see our Editorial Policies and the Editorial Policy Checklist.

Statistics

For all statistical analyses, confirm that the following items are present in the figure legend, table legend, main text, or Methods section.

n/a	Confirmed	
☐	☒	The exact sample size (*n*) for each experimental group/condition, given as a discrete number and unit of measurement
☐	☒	A statement on whether measurements were taken from distinct samples or whether the same sample was measured repeatedly
☒	☐	The statistical test(s) used AND whether they are one- or two-sided<br>*Only common tests should be described solely by name; describe more complex techniques in the Methods section.*
☒	☐	A description of all covariates tested
☒	☐	A description of any assumptions or corrections, such as tests of normality and adjustment for multiple comparisons
☐	☒	A full description of the statistical parameters including central tendency (e.g. means) or other basic estimates (e.g. regression coefficient) AND variation (e.g. standard deviation) or associated estimates of uncertainty (e.g. confidence intervals)
☒	☐	For null hypothesis testing, the test statistic (e.g. *F*, *t*, *r*) with confidence intervals, effect sizes, degrees of freedom and *P* value noted<br>*Give P values as exact values whenever suitable.*
☒	☐	For Bayesian analysis, information on the choice of priors and Markov chain Monte Carlo settings
☒	☐	For hierarchical and complex designs, identification of the appropriate level for tests and full reporting of outcomes
☒	☐	Estimates of effect sizes (e.g. Cohen's *d*, Pearson's *r*), indicating how they were calculated

Our web collection on statistics for biologists contains articles on many of the points above.

Software and code

Policy information about availability of computer code

Data collection	Clinical, pathology and sequencing data files are collected and subsequently analysed by the MTB portal by using appropriate secure transfer protocols and data access security measures (not disclosed here). Additional regulation details can be found in the manuscript.
Data analysis	MTBP variant annotation and interpretation combines multiple resources following expert-consensus criteria as described in detail in the manuscript. The analytical pipeline to interpret the data is built in Python (>3.0), with versions spanning from that available for the patient n=1 (pipeline v1.0) to that available for the patient n=500 of this present cohort (pipeline v4.8), and aggregating several publicly available bioinformatics tools (TransVar v2.5 and VEP v94-101) and knowledgebases (ClinVar, BRCA-Exchange, OncoKB, CIViC CGI, 1000g, gnomAD -- with database releases corresponding to those available at the moment of registering each patient of the present cohort - i.e. January 2019 to January 2021). More details and references can be found in the manuscript.

For manuscripts utilizing custom algorithms or software that are central to the research but not yet described in published literature, software must be made available to editors and reviewers. We strongly encourage code deposition in a community repository (e.g. GitHub). See the Nature Research guidelines for submitting code & software for further information.

Data

Policy information about availability of data

All manuscripts must include a data availability statement. This statement should provide the following information, where applicable:

- Accession codes, unique identifiers, or web links for publicly available datasets
- A list of figures that have associated raw data
- A description of any restrictions on data availability

Sequencing data has been deposited at the European Genome–phenome Archive (EGA), which is hosted by the European Bioinformatics Institute and the Centre

for Genomic Regulation. Due to patient privacy constraints, the data is under controlled access and available upon reasonable request to the Cancer Core Europe Basket of Baskets Data Access Committee (bob@vhio.net) based on EGA terms (see accession number EGAS00001005893 for further details).

Field-specific reporting

Please select the one below that is the best fit for your research. If you are not sure, read the appropriate sections before making your selection.

☒ Life sciences ☐ Behavioural & social sciences ☐ Ecological, evolutionary & environmental sciences

For a reference copy of the document with all sections, see nature.com/documents/nr-reporting-summary-flat.pdf

Life sciences study design

All studies must disclose on these points even when the disclosure is negative.

Sample size	This paper describes the deployment of an academic clinical decision support system across a network of European Cancer Centers. To illustrate its results, we also present the results (aggregated at the cohort level) of using the system in a prospective, consecutive cohort of cancer patients pre-registered to the Basket of Baskets trial (NCT03767075) during two years, summing up a total of 500 evaluated tumors. Such number of tumors and time span are considered large enough to illustrate the performance of the system and the experience of using it, which is the aim of the present manuscript, and thus no other sample size calculations apply here.
Data exclusions	No sample of the aforementioned cohort was excluded in the results presented here
Replication	Sequencing results were interpreted using the MTB portal analytical framework developed under the Cancer Core Europe umbrella. The MTBP analytical framework was systematically applied by an automated workflow across the whole cohort presented here. The pipeline code and the resources employed to annotate the patients' results are under version control in the corresponding software and data repositories employed by the system, respectively. Before releasing any code and/or data update, the system is tested in a stage server by semi-automatic workflows based on predefined test input data according to our standard operating procedures.
Randomization	This manuscript includes the results of using the MTB portal in a prospective, consecutive cohort of cancer patients pre-registered to the Basket of Baskets trial (NCT03767075) during two years. The Basket of Baskets allocates tumors with pre-defined molecular profiles to treatment arms testing different targeted drugs and immune therapies (no patient randomization). However, this manuscript describes the results of interpreting the genomics data (aggregated at the cohort level), but no clinical outcomes connected to the trial are reported here.
Blinding	This manuscript includes the results of using the MTB portal in a prospective, consecutive cohort of cancer patients pre-registered to the Basket of Baskets trial (NCT03767075) during two years; the trial arms opened during this time use no blinding, but note that (as stated in the previous section), the paper describe the results of interpreting the genomics data (aggregated at the cohort level), but no clinical outcomes connected to the trial are reported here.

Reporting for specific materials, systems and methods

We require information from authors about some types of materials, experimental systems and methods used in many studies. Here, indicate whether each material, system or method listed is relevant to your study. If you are not sure if a list item applies to your research, read the appropriate section before selecting a response.

Materials & experimental systems

n/a	Involved in the study	
☒	☐	Antibodies
☒	☐	Eukaryotic cell lines
☒	☐	Palaeontology and archaeology
☒	☐	Animals and other organisms
☐	☒	Human research participants
☐	☒	Clinical data
☒	☐	Dual use research of concern

Methods

n/a	Involved in the study	
☒	☐	ChIP-seq
☒	☐	Flow cytometry
☒	☐	MRI-based neuroimaging

Human research participants

Policy information about studies involving human research participants

Population characteristics	Results of interpreting the NGS tumor data of a total of 500 patients (median 59y, 60% female) with advanced solid cancers pre-registered to the Basket of Baskets (NCT03767075) study during a 2-years period are presented in this manuscript (results aggregated at the cohort level; characteristics of the cohort detailed in Table 1 of the manuscript).
Recruitment	Patients pre-registered in the Basket of Baskets trial (NCT03767075) during 2 years. Further inclusion/exclusion trial criteria available at https://clinicaltrials.gov/ct2/show/NCT03767075 . All included patients signed the corresponding informed consent for molecular profiling and (when pertinent) trial participation (more details in the manuscript).

Ethics oversight	The Vall d'Hebron Institute of Oncology (VHIO) is the sponsor of the Basket of Baskets trial. The protocol was submitted through the Voluntary Harmonization Procedure (VHP) and approved by the Medicines & Healthcare products Regulatory Agency (MHRA) in the UK. Subsequently, the competent authorities in Spain (Agencia Española de Medicamentos y Productos Sanitarios - AEMPS), France (Agence nationale de sécurité du medicament et des produits de santé - ANSM), Germany (Bundesinstitut für Impfstoffe und biomedizinische Arzneimittel), The Netherlands (Centrale Commissie Mensgebonden Onderzoek - CCMO), and Sweden (Läkemedelsverket) provided local approval. Ethics Committee approvals have been obtained in Spain, UK, France, The Netherlands and Sweden, under the EduraCT project number 2018-005108-89. More details in the manuscript.

Note that full information on the approval of the study protocol must also be provided in the manuscript.

Clinical data

Policy information about clinical studies

All manuscripts should comply with the ICMJE guidelines for publication of clinical research and a completed CONSORT checklist must be included with all submissions.

Clinical trial registration	NCT03767075
Study protocol	The manuscript reports general genomic findings of patients preregistered to the Basket of Baskets trial https://clinicaltrials.gov/ct2/show/NCT03767075 during a 2-years period
Data collection	Patient clinical and tumor pathological information (e.g. sex, age, diagnosis, tumor sample characteristics and patient's previous treatments) are gathered by members of the clinical teams in each patient's medical institution in pseudo-anonymised electronic case report forms (ALEA system, https://www.aleaclinical.eu/). Molecular assay results (cancer gene NGS panels) are provided by the institutional facilities of Cancer Core Europe and commercial laboratories as required by each Basket of Baskets study module. Data transfer, storage and sharing is performed by the MTB portal by implementing a number of measures complying with Cancer Core Europe ethical/legal/cibersecurity framework, as described in the manuscript. This manuscript includes the results of interpreting the data of 500 consecutive patients preregistered to the Basket of Baskets trial (NCT03767075) during a 2-years period (January 2019 to January 2021).
Outcomes	The Global objective of this Basket of Basket study is to evaluate the antitumor activity of each matched therapies that will be evaluated through the study in small molecularly selected populations. The primary objective of module 1 is to determine the overall response rate by RECIST 1.1 after 3 weeks of treatment with atezolizumab in several arms selected according to predefined molecular alterations. Secondary outcomes include: mean progression free survival (PFS by RECIST 1.1; Time Frame: through study completion); progression Free Survival (PFS by RECIST 1.1; Time Frame: 6 months); mean overall survival; Time Frame: through study completion). However, note that this manuscript present the results of interpreting the NGS data of 500 consecutive tumors registered for the Basket of Baskets study (aggregated at the cohort level), but this manuscript does not report outcomes of the clinical interventions associated to the trial study.
